# Synthesis of *Ziziphus spina-christi* (Jujube) Root Methanol Extract Loaded Functionalized Silver Nanoparticle (ZS-Ag-NPs); Physiochemical Characterization and Effect of ZS-Ag-NPs on Adipocyte Maturation, Adipokine and Vascular Smooth Muscle Cell Interaction

**DOI:** 10.3390/nano11102563

**Published:** 2021-09-29

**Authors:** Abu ElGasim Ahmed Yagoub, Ghedeir Muslem Alshammari, Pandurangan Subash-Babu, Mohammed Awad alkareem Mohammed, Mohammed Abdo Yahya, Aesha Ibrahim Alhosain

**Affiliations:** Department of Food Science and Nutrition, College of Food and Agricultural Sciences, King Saud University, P.O. Box 2460, Riyadh 11451, Saudi Arabia; amohammed4@ksu.edu.sa (A.E.A.Y.); sbpandurangan@ksu.edu.sa (P.S.-B.); 442106434@student.ksu.edu.sa (M.A.a.M.); 441106332@student.ksu.edu.sa (M.A.Y.); 439204473@student.ksu.edu.sa (A.I.A.)

**Keywords:** *Ziziphus spina-christi* (L.), jujube, silver nanoparticle, adipocytes, angiogenesis

## Abstract

In this research, a simple, green approach was employed to synthesize silver nanoparticles with the aid of *Ziziphus spina-christi* (L.) methanol root extract, which can act as a reducing, capping agent to treat obesity and inflammation. Globally, *Ziziphus spina-christi* (Jujube) root is used in traditional therapy as a lipolysis promoter. GC-MS results confirmed the availability of kaempferol (flavonol), cannabinol and indole-3-carboxylic acid in *Ziziphus spina-christi* root methanol extract (ZSE). ZSE silver nanoparticles (ZS-Ag-NPs) were synthesized and their effect on mitochondrial fatty acid oxidation capacity and adipokine levels in maturing adipocytes were analyzed. Maturing adipocytes treated with 0.4 µg/dL of ZSE and ZS-Ag-NPs significantly reduced the lipid content in adipocytes by 64% and 82%, respectively. In addition, lipolysis-related genes such as LPL (1.9 fold), HSL (2.3 fold), PGC-1α (3 fold), UCP-1 (4.1 fold), PRDM16 (2 fold) and PPARα (2.7 fold) increased significantly in ZS-Ag-NPs treated maturing adipocytes. The ZS-Ag-NPs treatment significantly decreased insulin resistance and metabolic inflammation-related LTB4-R, TNF-α, IL-4 and STAT-6 mRNA levels. Mitochondrial thermogenesis stimulating capacity of ZS-Ag-NPs was further confirmed by the significantly enhanced CREB-1 and AMPK protein levels in adipocytes. Furthermore, ZS-Ag-NPs treated adipokines (condition media, CM) were treated with human umbilical vein endothelial cells (HUVECs) to determine cytotoxicity and pro-inflammatory stimulus capacity. We found that ZS-Ag-NPs treated adipocyte CM effectively increased mRNA expression levels of the vascular endothelial cell growth factor (VEGF), and down-regulated oxidative stress (LPO, eNOS, and HO) and vascular cell inflammation (ICAM, VCAM, TNF-α, IL-1β, and NF-κB). In conclusion, ZS-Ag-NPs displayed an action at the molecular level in mitochondrial fatty acid oxidation, decreased adipokine secretion in adipocytes, and enhanced vascular endothelial cell growth. This molecular mechanical action of ZS-Ag-NPs reduced effectively obesity progressions and metabolic inflammatory pathogenesis associated with aging.

## 1. Introduction

Obesity is accompanied by excessive fat storage in adipose tissue and it serves as a highly active metabolic and endocrine organ. It stimulates or directly produces many pro-inflammatory cytokines and hormonal mediators together called adipokines [1]. Adipokines are positively associated with the progression of local and systemic inflammation end with chronic metabolic disorders [2]. Adiposity is accompanied by the overproduction of pro-inflammatory adipocytokines, which have an impact on vascular smooth muscle cells, such as vascular injury, vascular cellular senescence, and targeted organ damage [3]. Pro-inflammatory adipokines excreted by hypertrophic adipocytes, namely interleukin (IL)-1β, IL-6, nuclear factor kappa-light-chain-enhancer of activated B cells (NF-κB), and tumor necrosis factor (TNF)-α can exacerbate various metabolic and cardiovascular diseases [1,4]. However, few adipokines, such as adiponectin, exhibit anti-inflammatory properties with protective functions against atherogenesis and obesity-related diseases [3]. Overall, an imbalance in the production of pro-inflammatory and anti-inflammatory adipokines from the hypertrophic adipocyte results in the progression of irreversible multiple complications.

The discovery of a pharmacological agent for obesity is a challenging risk because adipogenesis is regulated by numerous transcription factors that are associated with an energy imbalance, whereby a long-term nutrients overload occurs. The cAMP pathway and AMP-activated protein kinase (AMPK) are recognized as sensors of cellular energy and are crucial to the inhibition of adipocyte development [5]. C/EBP-α is a central transcriptional adipogenic activator that is regulated by the cAMP-responsive element-binding protein (CREB). The binding of cAMP to the PKA regulatory subunit results in the release of a catalytic subunit, which can then phosphorylate its lipid metabolism-associated protein substrates, including AMPK in peripheral and subcutaneous adipocytes [6]. AMPK is activated during the depletion of the cellular ATP, which increases the AMP/ATP ratio and initiates metabolic and genetic events to restore ATP levels via fatty acid beta-oxidation in adipocytes [7]. 

Currently, most of the anti-obesity drugs have failed and fell into disrepute, either due to their ineffectiveness or adverse side effects [8]. Traditional medicinal plant-derived active metabolites, such as quercetin, catechin, curcumin, and tea polyphenols have been known to have lipid-lowering potentials with lower side effects, even though they failed to provide a multilevel anti-obesity potential [9]. Many of the natural products have rigorously restricted their development, because of their membrane permeability, surface properties, and bioavailability [10]. However, the synthesis of silver nanoparticles loaded with plant materials can functionalize the materials, reduce capture by the reticuloendothelial system, and increase the systemic circulation of nanomedicines [11]. Previously, the green synthesis of silver nanoparticles using isoorientin, a natural flavonoid, led to the formation of nanoparticles characterized by high stability after gastrointestinal digestion, low cytotoxicity and inhibition potentials against α- glucosidase and pancreatic lipase [12]. In addition, green synthesis of silver nanoparticles with cranberry powder [13], *Ficus palmate* leaves [14], and *Pisum sativum* L. [15] have been identified with more stabilized and enhanced anti-inflammatory, antioxidant, and antibacterial activities than their respective extracts alone. In this study, we synthesized *Ziziphus spina-christi* (Jujube) root-loaded silver nanoparticles (ZS-Ag-NPs) and used them to examine their effect on lipolysis, mitochondrial fatty acid oxidation capacity, and adipokine levels in maturing adipocytes. In addition, ZS-Ag-NPs treated adipokines (condition media) were used to analyze their effect on human umbilical vein endothelial cell’s (HUVEC) proliferation, oxidative stress, and inhibition of pro-inflammatory cytokines associated with cardiovascular diseases, angiogenesis, and ageing.

## 2. Materials and Methods

### 2.1. Extract Preparation and Composition Analysis

#### 2.1.1. Preparation of *Ziziphus spina-christi* (L.) (Jujube, Sidhr) Root Methanol Extract

*Ziziphus spina-christi* (L.) roots were obtained from Darfur, Sudan and identified by a taxonomist in King Saud University, Riyadh (A specimen sample is kept in Department Herbarium). The shade-dried *Ziziphus spina-christi* (L.) roots were crushed and suspended in 95% methanol at a solid-to-solvent ratio of 1:10 in a conical flask. After wrapping with aluminum foil, the flask was shaken for 6 h using a Wrist Action shaker (Burrell Scientific, Pittsburgh, PA, USA). The extractive was filtered (Whatman No. 1 filter paper), and then concentrated *in vacuo* using a rotary evaporator (HAHNVAPOR, HS-2005, Hahn Shin Sientific, Gimpo-si, Korea). The concentrated root extract (4.9 mg/mL) was kept for further use. 

#### 2.1.2. GC-MS Analysis of *Ziziphus spina-christi* Methanol Root Extract

The phytochemical content of *Z. spina-christi* methanol root extract was analyzed using an Agilent 7890A (Agilent Technologies, Santa Clara, CA, USA) gas chromatography (GC) coupled with a 5975C inert mass-spectrometer (MSD). The system was equipped with a DB-5MS GC column (30 m length, 0.25 mm inner diameter, and 0.25 µm film thickness), a Triple-Axis detector (MSD), and a 7693 automated liquid sampler. One milliliter of the extract was filtered through a 2 µm membrane filter. An aliquot (1 µL) of the extract was injected into the system. The injection temperature was 280 °C and the column temperature was 300 °C. Helium was used as the carrier gas with a flow rate of 1 mL/min. The electron ionization energy was 70 eV. 

### 2.2. Synthesis of Ziziphus spina-christi Root Methanol Extract Loaded Silver Nanoparticles

*Ziziphus spina-christi* root extract (ZSE) and loaded, silver nanoparticles (ZS-Ag-NPs) were synthesized by drop-wise addition of 10 mL of ZSE to 30 mL of different AgNO_3_ solutions (concentrations: 1.5, 3.0, 5.0, and 10.0 mM) in the dark at room temperature, with continuous stirring (200 rpm, 90 min). The pH of reaction mixtures was adjusted to 12 using a NaOH solution (dissolved in deionized water). At the end of the reaction, the mixtures acquired light yellowish-brown to brown colors, confirming the formation of ZS-Ag-NPs. The mixtures were then dried at 60 °C for 24 h to get nanoparticle pellets. The pellets were milled using clean mortar and pestle and kept in brown bottles for later use in characterization and biological studies. 

#### Characterization of Silver Nanoparticles (ZS-Ag-NPs)

To confirm the formation of ZS-Ag-NPs, the reduction of Ag^+^ ions was examined by a UV-visible spectrophotometer (UV-2450 double-beam, Shimadzu, Tokyo, Japan). The UV-visible spectra of the ZSE, and AgNO_3_ and ZS-Ag-NPs suspensions (1.5–10 mM) were measured at a wavelength range of 200–800 nm. The crystalline phase analysis of the synthesized nanoparticles was performed by measuring X-ray powder diffraction (XRD) patterns using a diffractometer (Bruker D8 Advance) equipped with a Cu-Kα radiation source (λ = 1.54 nm; 40 kV; 40 mA) and a diffracted beam monochromator. The scattered radiations were detected in the angular range of 10–90° (2θ) with a scan rate of 0.02°. Diffraction patterns of the extract, AgNO_3_, and ZS-Ag-NPs were compared with the JCPDS card database. Morphological images of samples were taken by a transmission electron microscope (TEM) (JEM-1011, JEOL Ltd., Tokyo, Japan) working at an acceleration voltage of 160 kV. The size distribution of ZS-Ag-NPs was determined by using a Zetasizer (HT Laser, ZEN3600 Malvern, Nano series, Instruments, Malvern, UK). The functional groups of the plant extract and ZS-Ag-NPs were analyzed by using a Nicolet 6700 Fourier-transform infrared (FT-IR) spectrometer (Waltham, MA, USA) at a wavenumber range of 500–4000 cm^−1^. 

### 2.3. Biology

#### 2.3.1. Chemicals

Human mesenchymal stem cells (hMSCs) and human umbilical vein endothelial cell lines (HUVECs) were obtained from American Type Culture Collection (ATCC, Manassas, VA, USA). Dulbecco’s modified Eagle medium (DMEM), trypsin, EDTA, and all cell culture materials were purchased from Gibco, Paisley, UK. Cell culture materials, such as fetal bovine serum and penicillin-streptomycin, were obtained from HyClone Laboratories, USA. MTT [3-(4,5-dimethylthiazol-2-yl)-2,5-diphenyltetrazolium bromide], ORO oil red’O), and Nile red were purchased from Sigma (St. Louis, MO, USA). Adipocyte differentiation factors such as insulin, rosiglitazone, dexamethasone (DEX), 3-isobutyl-1-methyl-xanthine (IBMX), and lipopolysaccharides (LPS) were purchased from Sigma (St. Louis, MO, USA). The cytokine-analyzing ELISA array kits were purchased from Qiagen (MEH004A, Qiagen, Hilden, Germany). The cDNA synthesis kit and SYBR Green PCR Master Mix were purchased from Qiagen, Hilden, Germany. All other chemicals related to the molecular biology experiment were purchased from Sigma-Aldrich (St. Louis, MO, USA).

Before starting cellular experiments, the nanoparticle powder was sterilized in a UV light for 10 min to eliminate any microbial contaminations would happened during storage. All nanoparticles dilutions for biological treatments were carried out freshly.

#### 2.3.2. hMSCs Culture and Adipocyte Differentiation

Human mesenchymal stem cells were cultured using Dulbecco’s modified Eagle medium (DMEM) containing 10% fetal bovine serum and 100 U/mL penicillin-streptomycin at 37 °C in a humidified 5% CO_2_ using an incubator. Cells were seeded in 24-well plates at a density of 2 × 10^4^ cells/well. The cells were grown, to reach 90% confluence, in DMEM/high glucose containing 10% FBS at 37 °C and 5% CO_2_ humidified air. Forty-eight hours after visual confluence (day 0), cells were replaced with adipocyte differentiation media (DMEM containing 10% FBS, 1 µM dexamethasone, 0.5 mM IBMX and 10 μg/mL insulin) for the next three days. On Day 3, cells were then cultured in adipogenesis maturation medium (DMEM containing 10% FBS and 10 μg/mL insulin) for two consecutive days. Subsequently, the cells were cultured in a maintenance medium (DMEM with 10% FBS) for six days, a fresh medium was replaced every two days. For all assays, cells cultured only in the maintenance medium were used as a control. 

#### 2.3.3. Cytotoxicity Analysis

Human mesenchymal stem cells (hMSCs) were induced to differentiate into adipocytes in 96-well culture plates (1 × 10^4^ cells/well) and allowed to adhere overnight in DMEM. After discarding the medium, a culture medium containing *Ziziphus spina-christi* root methanol extract (ZSE) or *Ziziphus spina-christi* root extract loaded silver nanoparticle (ZS-Ag-NPs) (0, 0.2, 0.4, 0.8, 1.6, and 3.2 µg/dL) was added to each well, and the cells were incubated for 24 h to 48 h; untreated cells were used as controls. After completion of the time, the cells were carefully washed with PBS, then a medium containing 5 mg/mL MTT (3-[4,5-dimethylthiazol-2-yl]-2,5-diphenyltetrazolium bromide) in DMEM was added to each plate well (i.e., 20 µL/well). The plates were incubated at 37 °C for an additional 4 h. At the end of incubation, the medium was removed, and the purple formazan produced was dissolved in 100 µL of DMSO. The absorbance of the solution was measured at 570 nm using a microplate reader (Thermo Scientific, Waltham, MA, USA). The cell proliferation (%) was calculated by the following equation: (the absorbance of the sample/the mean absorbance of the control) × 100.

#### 2.3.4. Experimental Design

The differentiated adipocytes (3rd day) were treated with different concentrations of ZSE, AgNO_3_, ZS-Ag-NPs (0.1, 0.2 and 0.4 µg/dL), and the reference drug, orlistat (6 µM), and maintained until day 14. The maintenance medium was replaced once in 3 days. After that, the effective dose of ZSE and ZS-Ag-NPs was selected according to the ability to inhibit lipid accumulation after 14 days [16]. In another set of experiments, the condition media of untreated adipocytes, the adipocytes treated with ZSE, AgNO_3_, ZS-Ag-NPs, and orlistat (6 µM) were collected on day 14.

#### 2.3.5. Interaction of ZS-Ag-NPs Treated Adipokines with HUVECs

To analyze the interaction of adipocytes and HUVECs, condition media of the untreated adipocytes, adipocytes treated with ZSE, AgNO_3_, ZS-Ag-NPs and orlistat (6 µM) were treated with HUVECs for 48 h, such as 50% condition medium and 50% normal growth medium (50:50). At the end of the experiment, the cells and the condition medium were collected and processed for the quantification of pro-inflammatory and atherogenesis and angiogenesis-related mRNA and protein levels.

#### 2.3.6. Oil Red’O and Nile Red Staining Analysis to Determine Lipid Accumulation Levels

Differentiated preadipocytes were maintained in 24-well plates and treated with ZSE, AgNO_3_, ZS-Ag-NPs (0.1, 0.2 and 0.4 µg/dL), and orlistat (6 µM) and incubated in the CO_2_ incubator for 14 days. The maintenance media were changed once in 3 days. After 14 days, cells were washed twice with PBS and fixed with 4% (*v*/*v*) paraformaldehyde for 1 h at room temperature. Thereafter, the treated cells were subsequently washed with PBS and isopropanol 60% (*v*/*v*) and left to dry. Then, the treated cells were stained with a filtered 0.5% (*v*/*v*) Oil red’O solution (60% isopropanol and 40% water) for 1 h. Then, the Oil red’O staining solution was removed, and the plates were rinsed with distilled water thrice and left to dry. The stained lipid droplets were viewed at 20× magnification on a microscope and were photographed. After the image analysis, the stained cells were dried overnight and the oil stains were dissolved with isopropanol to measure the absorbance at 520 nm.

For the Nile red staining assay, a stock solution containing 5 mg of Nile red dissolved in 1 mL of 100% acetone was used. After 14 days of ZSE, AgNO_3_ and ZS-Ag-NPs (0.4 µg/dL) treatments, preadipocytes were fixed with formaldehyde, then stained with 200 μL of fluorescence Nile red (working solution: 6 μL of stock Nile red dissolved in 1 mL of 40% isopropanol) for 30 min at room temperature. Then, the stained cells were analyzed using an inverted fluorescence microscope and photographs were taken immediately using a fluorescent microscope.

#### 2.3.7. Analysis of Mitochondrial Membrane Potential Using JC-1 Staining

The loss of the mitochondrial membrane potential (Δψm) was determined using a JC-1 probe, which exists in monomeric form predominantly in cells with depolarized mitochondria and fluorescence green. Cells with polarized mitochondria containing predominantly JC-1 aggregates emitted a reddish-orange fluorescence. Untreated adipocytes and adipocytes treated with ZSE, AgNO_3_, and ZS-Ag-NPs (0.4 µg/dL) were incubated with 5 mM of JC-1 for 10 min at 37 °C. Then, the cells were washed with a JC-1 washing solution and the acquired signals were analyzed with a fluorescent microscope.

#### 2.3.8. Propidium Iodide Staining for Nuclear Damage Analysis in HUVECs

Cell apoptosis was quantified using the propidium iodide (PI) (Sigma Chemicals, St. Louis, MO, USA). ZS-Ag-NPs treated adipocyte condition media treated HUVECs (1 × 10^5^/well) were plated in a 24-well plate and incubated with ZSE, AgNO_3,_ ZS-Ag-NPs (0.4 µg/dL) and the vehicle control for 48 h. Following that, the cells were incubated with 5 μL of PI in the dark for 15 min at room temperature (RT). The cells were analyzed by an inverted microscope to identify the nuclear damage or chromatin condensation levels and photographs were taken and recorded.

#### 2.3.9. Quantitative Polymerase Chain Reaction (qPCR) Analysis

Vehicle control, ZSE, ZS-Ag-NPs (0.4 μg/dL) and orlistat (6 µM) treated maturing adipocyte and HUVECs total RNA and cDNA were synthesized using the Fastlane^®^ Cell cDNA kit and a semi-automative qPCR instrument (Applied Biosystems, Foster City, CA, USA). Adipocyte hyperplasia and hypertrophy (C/EBPα, PPARγ, HSL, LPL, SREBP-1c and FABP-4); fatty acid oxidation and energy expenditure (Adiponectin-R1, PPARγC1α, UCP-1 and PRDM16) in adipocytes were analyzed. In HUVECs, mRNA levels of the oxidative stress (LPO, eNOS, and HO), vascular inflammation (ICAM, VCAM, TNF-α, IL-1β, NF-κB), and the vascular cell growth factor (VEGF) were quantified against the reference gene, β-actin, according to the reported method [17]. The amplification values (ΔCt) were calculated based on the difference between the Ct value of treated maturing adipocytes and the Ct value of the control. The expression of the 2^−ΔΔCt^ values was used to plot the gene expressions.

#### 2.3.10. Quantification of Protein Using ELISA

The amount of metabolic inflammation, insulin resistance and fatty acid metabolism deregulating markers, such as CREB-1, AMPK, NF-Κb, and TNF-α (in adipocytes) were analyzed in vehicle control, ZSE and ZS-Ag-NPs (0.4 μg/dL) treated cells using high-sensitivity ELISA-kits (Quantikine, R&D Systems, Minneapolis, MN, USA). This assay does not distinguish between soluble and receptor-bound proteins and thus it gives a measure of the total concentration of inflammatory mediator proteins. The values were expressed as pg/mg protein. 

### 2.4. Statistical Analysis

All data obtained from experiments were statistically evaluated using SPSS/28.5 software package. The data were analyzed by the one-way analysis of variance (ANOVA) and followed by Tukey’s multiple comparison test. All results were expressed as mean ± SD (*n* = 6). *p* values were considered significant at <0.05.

## 3. Results

### 3.1. GC-MS Analysis of Ziziphus spina-christi Root Methanol Extract 

GC-MS analysis of *Ziziphus spina-christi* methanol root extract revealed phytochemicals with considerable amounts, such as indole-3-carboxylic acid, 5-methoxy-2-methyl-1-(3-methylphenyl)-, ethyl ester (19.02% of the total peak area), benzeneacetonitrile, 4-hydroxy- (18.04% of the total peak area), 2,4,6-Cycloheptatrien-1-one, 2-Coumaranone (9.28% of the total peak area), and 3,3-Diphenyl-1-indanone (9.16% of the total peak area). The GC-MS profile of the root extract detected compounds/derivatives with potential biological and pharmacological activities. For instance, indole-3-carboxylic acid, 5-methoxy-2-methyl-1-(3-methylphenyl)-, ethyl ester, Kaempferol (flavonol), Cannabinol (3.41% of the total peak area), 3,3-Diphenyl-1-indanone and 1,4- Phthalazinedione, 2,3-dihydro-6-nitro-, phenol, 2,2′-methylenebis[6-(1,1-dimethylethyl)-4-methyl (5.22% of the total peak area), and undecane (5.15% of the total peak area) (Table 1, Figure S1). 

### 3.2. Characterization of Silver Nanoparticles

UV–vis spectrophotometry is a powerful, sensitive tool for the initial recognition of the synthesis of nanoparticles prepared using different AgNO_3_ concentrations: 1.5–10 mM (Figure S2a). Figure S2b shows the UV-vis absorption spectra of the *Z. spina-chrisiti* root extract, AgNO_3_, and ZS-Ag-NPs. All silver nanoparticle samples showed maximum absorption bands with varying intensities, which might have resulted from the localized surface plasmon resonance phenomenon [36]. This phenomenon is ascertained with the bio-reduction of Ag+ cations into silver particles [37]. As seen, increasing the concentration of AgNO_3_ from 1.5 mM to 10 mM led to a red-shift of the plasmon band of AgNO_3_ (from <400 nm) after the synthesis of ZS-Ag-NPs. This was probably ascribed to the higher nucleation rate that happened during the reduction process [38]. The surface plasmon peak of 5 mM ZS-Ag-NPs has occurred at ~421 nm and 10 mM ZS-Ag-NPs at ~418 nm. However, the change in the maximum absorption band of silver nanoparticle solutions was seen as a color change. The UV-vis spectra of 1.5 and 3 mM ZS-Ag-NPs were broader with lower intensities than 5 and 10 mM ZS-Ag-NPs, indicating the formation of destabilized nanoparticle aggregates [39]. Accordingly, 5 and 10 mM ZS-Ag-NPs were prone to further characterization analyses. 

In addition, the functional groups present on nanoparticle surfaces, identified by FT-IR spectroscopy, are responsible for the reduction and capping reactions during the ZS-Ag-NPs biosynthesis. The FT-IR spectra of the root extract revealed a broad, strong peak at 3440 cm^−1^, corresponding to the stretching vibration of the phenolic and alcoholic O–H groups. This peak was reduced to 3429 and 3428 cm^−1^ in 5 and 10 mM ZS-Ag-NPs, possibly due to the interaction of O-H groups with silver cations to synthesize nanoparticles [40]. A weaker peak occurred at a frequency of 2918 cm^−1^ of ZS-Ag-NPs was assigned to the C-H stretching vibrations of the aliphatic groups [41]. A peak at 2110 cm^−1^ was assigned to alkyne groups present in biomolecules of the extract [42], which appeared as weaker peaks at 2340 cm^−1^ in ZS-Ag-NPs. A strong absorption peak that occurred at 1640 cm^−1^ in the root extract spectra was mainly related to amide I asymmetric stretching vibrations of C=O, coupled with little in-plane N-H bending [38]. This absorption peak shifted to 1627 and 1629 cm^−1^, with weaker intensities, in 5 and 10 mM ZS-Ag-NPs, indicating bonding of Ag^+^ cations with C=O amide groups. The peaks at 1384 and 1385 cm^−1^ were assigned to the stretching vibration of the nitro group N=O, which could be formed by the oxidation of the N-H_2_ group of amine and the reduction of silver cation into metal silver [43]. The FT-IR spectra of the extract showed a weaker absorption peak at 1019 cm^−1^, corresponding to C–O–C and C–O–H stretching vibrations of ether [44], which was disappeared in ZS-Ag-NPs spectra. Absorption bands at 1063 and 1077 cm^−1^ were observed in 5 and 10 mM ZS-Ag-NPs, possibly originated as a result of the C–N stretching vibration of aromatic and aliphatic amines of flavanones or terpenoids [41,42]. The weaker peaks at 601, 790, and 834 cm^−1^ were attributed to the aromatic C-H bending of phenolics in the extract and silver nanoparticles [45]. Absorption bands at 380–580 cm^−1^ observed in spectra of the root extract and ZS-Ag-NPs could be related to aromatic nitrile [40] (Figure S2c). 

**Figure 1:**
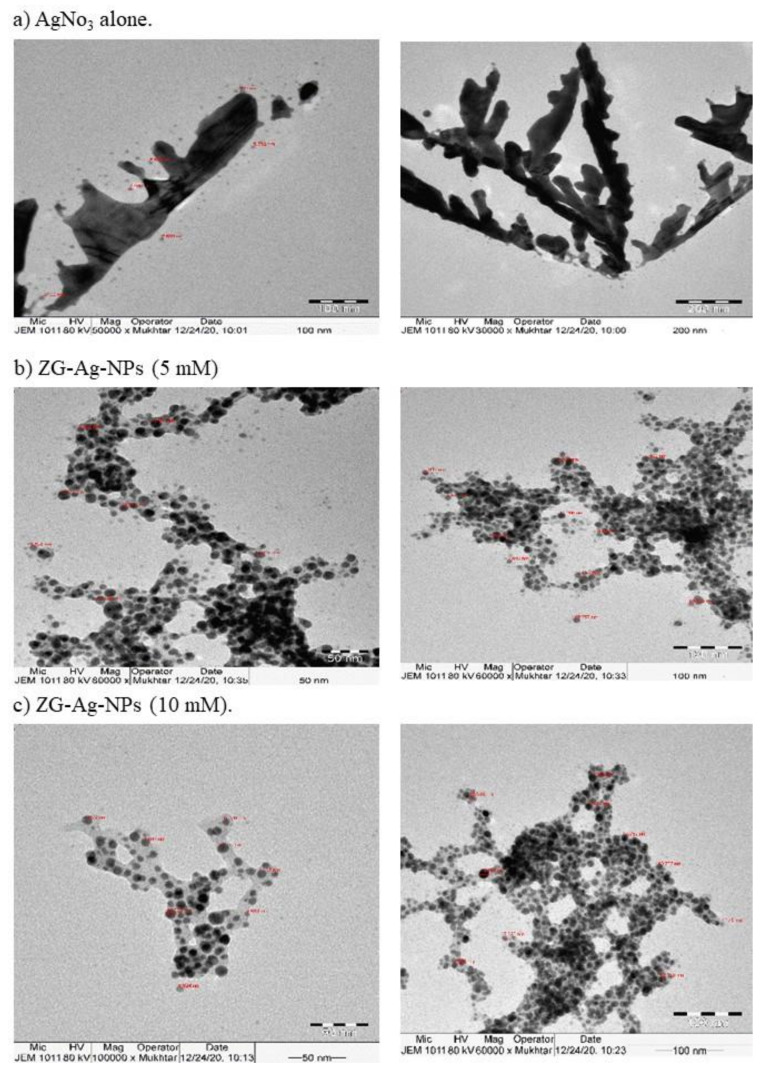
TEM images of AgNO_3_ (**a**), 5 mM ZS-Ag-NPs (**b**), and 10 mM ZS-Ag-NPs (**c**).

XRD spectra of 10 mM ZS-Ag-NPs showed five peaks at 32.20, 38.11, 46.16, 54.77, 57.38, and 67.37°, corresponded to lattice phases (110), (111), (200), (211), (211), and (200) reported in the Joint Committee on Powder Diffraction Standards (JCPDS card No. 00-076-1393) (Figure S3a–c). These lattice phases were indexed to face-centered cubic structures of spherical-shaped silver oxide nanoparticles [37,45]. In the case of 5 mM ZS-Ag-NPs, four major peaks were observed at positions that conform to the Miller indices of silver oxide, specifically 29.20° (100), 31.80° (110), 37.91° (111), and 77.11° (311) [46]. The existence of the XRD characteristic peaks of the face-centered cubic crystal structure of metallic silver, 44° (200), 64° (220), and 82° (222) (JCPDS card No. 04-0783) in the spectra of AgNO_3_ and 5 and 10 mM ZS-Ag-NPs (Figure S3a–c) was additional evidence that confirmed the crystallographic structure of ZS-Ag-NPs [47,48]. Moreover, the diffraction peaks of 10 mM ZS-Ag-NPs were relatively clear, with moderate miscellaneous peaks compared to those of the 5 mM ZS-Ag-NPs, suggesting good purity and size stability of 10 mM ZS-Ag-NPs. The average particle size of AgNO_3_ and 5 and 10 mM ZS-Ag-NPs was 6.69, 10.97, and 14.61 nm, respectively (Figure 1a–c). Silver oxide nanoparticles with different sizes (10–150 nm) have been biosynthesized earlier [39,40,49]. As seen, AgNO_3_ had spherical-shaped, agglomerated particles (Figure 1a), while ZS-Ag-NPs showed spherical-shaped, well-dispersed particles (Figure 1b, c). These observations were in line with the shape of bands in the UV-visible spectra. The size distribution by the intensity of 10 mM ZS-Ag-NPs showed a peak with most particles fell in the size range of 100–1000 nm, indicating a good dispersion (polysydisperse index, PdI = 0.521). In the case of 5 mM NPs, the particle size distribution (PdI = 0.262) fell partly between 100–1000 nm, with some particles located below 100 nm (Figure S4a–c). The size distribution data ascertained the stability of ZS-Ag-NPs colloidal solutions, which conformed to the results of UV-vis spectrophotometer and TEM. Because of its good dispersion and stability, the 10 mM ZS-Ag-NPs sample was used for the subsequent biological experiments. 

### 3.3. Cytotoxic Effects of ZSE and ZS-Ag-NPs on hMSCs, Preadipocytes and HUVECs 

**Figure 2:**
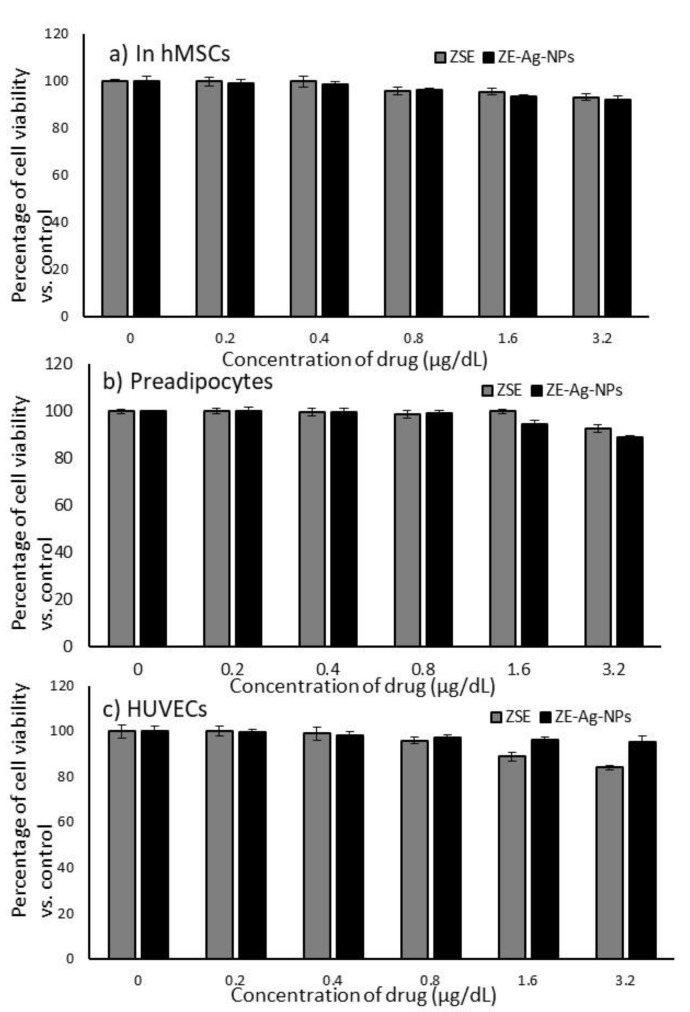
In vitro cytotoxic effects of *Ziziphus spina* (jujube) root methanol extract (ZSE) and *Ziziphus spina* (Jujube) root loaded silver nanoparticles (ZS-Ag-NPs) on hMSCs (**a**), preadipocytes (**b**), and HUVECs (**c**) after 48 h. Each value is a mean ± SD (*n* = 6).

We used different concentration doses (0, 0.2, 0.4, 0.8, 1.6, and 3.2 µg/dL) of ZSE and ZS-Ag-NPs to determine the cell viability inhibitory potential after 24 h and 48 h. The results revealed that minimum percentages of cell death in hMSCs, preadipocytes, and HUVECs treated with ZSE and ZS-Ag-NPs were observed even when using a higher dose (3.2 µg/dL) (Figure 2). After 48 h, 8% of cell death was observed in hMSCs (Figure 2a); 11% of cell death was observed in preadipocytes (Figure 2b), and 5% of cell death was observed in HUVECs (Figure 2c). The observed percentage of cell inhibition was not significant and not reached the IC_25_ values range. 

### 3.4. Dose Determination Based on Lipid Accumulation Inhibitory Potential Using Oil Red’O Staining Analysis

**Figure 3:**
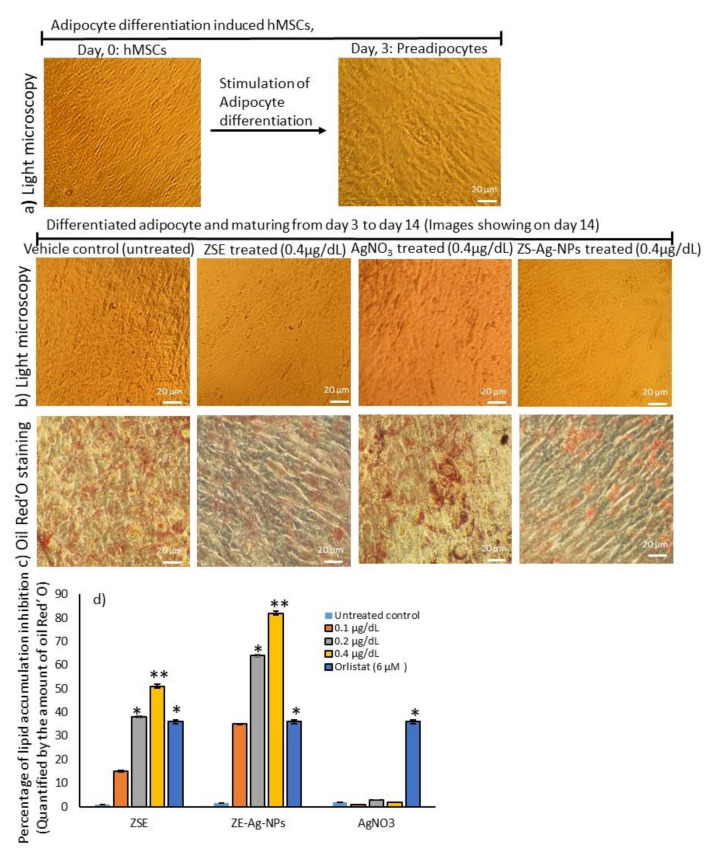
Images of adipocyte differentiation (**a**) in 3 days. Images of light microscopy (**b**), lipid accumulation by oil red’O staining (**c**), and the lipid inhibition percentage as quantified by oil red’O staining (**d**) of the effect of *Ziziphus spina* (jujube) root methanol extract (ZSE) and *Ziziphus spina* (Jujube) root loaded silver nanoparticle (ZS-Ag-NPs) on the adipocyte maturation after 14 days. Each value is a mean ± SD (*n* = 6). * significant at *p* ≤ 0.05 and ** highly significant at *p* ≤ 0.001, by comparison with the vehicle control.

In this study, ZSE and ZS-Ag-NPs at concentrations of 0.1, 0.2, and 0.4 μg/dL were selected for use in treating maturing adipocytes, aiming to assess their lipid accumulation inhibition potentials as per the experimental protocol. The differentiation of hMSCs to preadipocytes was proved by morphological images captured by a light microscope (Figure 3a). After 14 days, the light microscope images (Figure 3b) and oil red’O staining images (Figure 3c) disclosed an effective reduction in lipid accumulation in maturing adipocytes after a dose of 0.4 μg/dL of AgNO3, ZSE and ZS-Ag-NPs compared with the vehicle control. The results illustrated that the lipid accumulation was decreased significantly (*p* ≤ 0.001) by 82% and 64% after treating maturing adipocytes with ZS-Ag-NPs at doses of 0.4 μg/dL and 0.2 μg/dL, respectively, compared with the vehicle control (Figure 3d). The observed inhibitory effect of ZS-Ag-NPs on lipid accumulation was significantly higher when compared with that of ZSE treatment, that is, 0.4 μg/dL of ZS-Ag-NPs had an inhibitory effect of 82% versus 51% for ZSE. In comparison with ZSE and ZS-Ag-NPs doses of 0.4 μg/dL, 6 μM dose of orlistat decreased 36% of lipid accumulation. Compared to ZSE and ZS-Ag-NPs, AgNO_3_ did not show notable changes in adipocyte maturation or lipid accumulation inhibition, indicating that AgNO_3_ might lack an anti-obesity activity.

### 3.5. Determination of Hypertrophic Adipocytes Using Nile Red Fluorescence Staining Analysis

**Figure 4:**
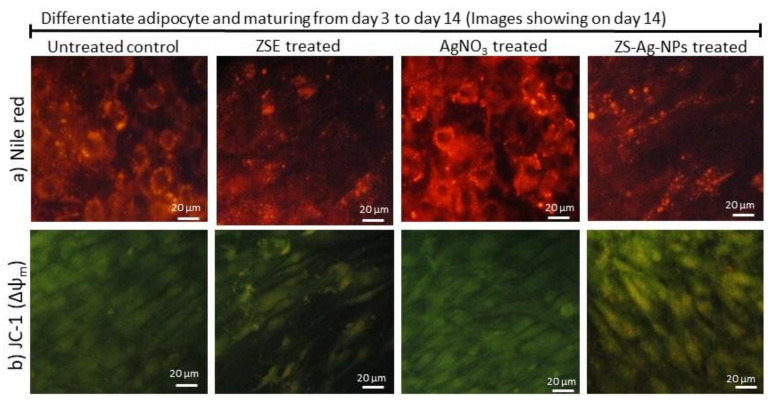
Nile red (**a**) and JC-1 staining (**b**) images of the vehicle control, ZSE, AgNO_3_, and ZS-Ag-NPs treated maturing adipocytes, after 14 days.

The Nile red analysis corroborated the occurrence of hypertrophy with high lipid fluorescence in untreated adipocytes after 14 days. Adipocytes treated with AgNO_3_, ZSE, and ZS-Ag-NPs at doses of 0.2 and 0.4 µg/dL significantly decreased (*p* ≤ 0.001) the lipid accumulation and adipocyte hypertrophy (Figure 4a). Interestingly, a 6 µM dose of orlistat did not produce significant inhibition against adipocyte hypertrophy, showing a lower effect than the dose of 0.4 µg/dL of ZSE (*p* ≤ 0.05). 

### 3.6. Mitochondrial Membrane Potential (JC-1) and Oxidative Capacity Analysis

Mitochondrial membrane potential (Δψm) is predicted the mitochondrial oxidative capacity for fatty acid energy metabolism. Figure 4b shows the JC-1 staining images of the vehicle control, AgNO_3_, ZSE, and ZS-Ag-NPs treated groups. The merged images of the JC-1 dye depicted red and green signals, corresponding to J-aggregates and monomeric forms, respectively. As seen, the dose of 0.4 µg/dL of ZSE and ZS-Ag-NPs led to linear and spindle-shaped adipocytes with high J-aggregates, which indicated higher mitochondrial efficiency on thermogenesis. In AgNO_3_ or ZSE treated maturing adipocytes, fewer J-aggregates were noticed, which were led to a lower mitochondrial efficiency on thermogenesis.

In Nile red staining, vehicle control shows hypertrophic adipocytes directly propositional to triglyceride storage. However, ZS-Ag-NPs treatment shows linear-shaped adipocytes with fewer lipid accumulation and fluorescent staining. ZS-Ag-NPs treated cells show the highest control on the adipocyte maturation compared with ZSE. AgNO_3_ alone did not reveal inhibition of adipocyte maturation.

JC-1 fluorescence images, merged images of the red and green signals of the dye correspond to J-aggregates and monomeric forms, respectively. Fewer J-aggregates are observed in the vehicle control. High J-aggregates, representing active mitochondria (high MMP, Δψ_m_), are seen in ZS-Ag-NPs and ZSE treated adipocytes.

### 3.7. Adipogenesis, Mitochondrial Thermogenesis, and Inflammatory Gene Expression Analysis in ZSE and ZS-Ag-NPs Treated Adipocytes

**Figure 5:**
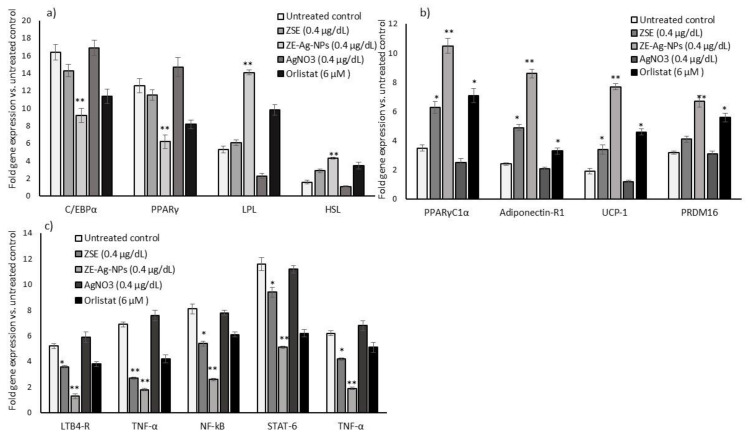
Effect of ZSE, AgNO_3_, ZS-Ag-NPs, and orlistat on adipogenesis (**a**), mitochondrial fatty acid oxidation, and thermogenesis (**b**) and adipokines responsible for metabolic inflammation (**c**) related gene expression levels in maturing adipocytes after 14 days. Each value is a mean ± SD (*n* = 6). * significant at *p* ≤ 0.05 and ** highly significant at *p* ≤ 0.001, by comparison with the vehicle control.

In comparison with the vehicle control or orlistat, maturing adipocytes treated with ZSE and ZS-Ag-NPs (0.4 µg/dL) for 14 days led to a significant (*p* ≤ 0.001) decrease in adipocytes hyperplasia related C/EBPα (0.6 fold), PPARγ (0.3 fold), and an increase in lipoprotein lipase (1.9 fold) and hormone-sensitive lipase (2.3 fold) mRNA expression levels (Figure 5a). Simultaneously, ZSE and ZS-Ag-NPs treatments of maturing adipocytes increased significantly adipocyte mitochondrial efficiency-related mRNAs, namely PPARγC1α (3 fold), Adiponectin-R1 (3.6 fold), UCP-1 (4.1 fold) and PRDM16 (2.1 fold) (Figure 5b). The metabolic inflammation-related genes, such as LTB4-R, TNF-α, NF-kB, STAT-6, and TNF-α expressions were decreased in ZS-Ag-NPs (0.4 µg/dL) treated maturing adipocytes compared with ZSE or orlistat treated groups (Figure 5c). 

### 3.8. Protein Levels in Adipocyte’s Stromal Vascular Fraction (SVF)

**Figure 6:**
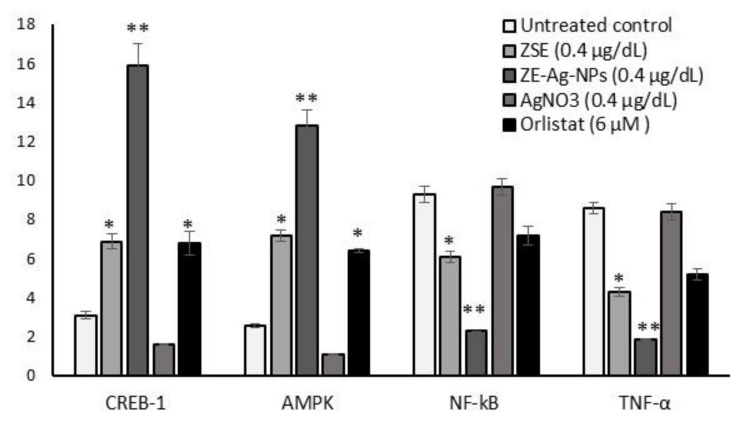
Quantification of adipocyte oxidative metabolism and metabolic inflammation-related protein levels in ZSE, AgNO_3_, ZS-Ag-NPs and orlistat treated maturing adipocytes (14 days)) using the ELISA method. Each value is a mean ± SD (*n* = 6). * significant at *p* ≤ 0.05 and ** highly significant at *p* ≤ 0.001, by comparison with the vehicle control.

The results of mitochondrial thermogenesis (CREB-1 and AMPK) and insulin resistance (NF-kB and TNF-α) related protein expression levels of ZSE and ZS-Ag-NPs (0.4 µg/dL) treated adipocytes are shown in Figure 6. In comparison with ZSE or orlistat treatments, CREB-1 and AMPK levels increased significantly (*p* ≤ 0.001), and NF-kB and TNF-α levels decreased after 14 days of ZSE and ZS-Ag-NPs treatments. 

### 3.9. Effect of ZSE and ZS-Ag-NPs Treated Adipocyte Condition Media on HUVECs Nuclear Damage and Angiogenesis Potential

#### 3.9.1. Light Microscopy, Propidium Iodide Staining for Morphology and JC-1 Staining for Mitochondrial Membrane Potential (Δψm) Analysis

**Figure 7:**
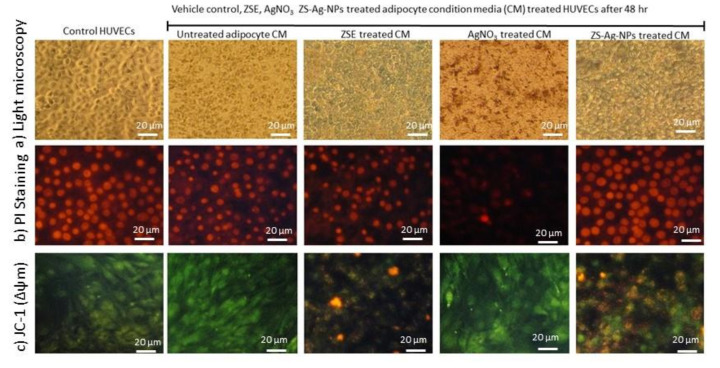
Analysis of the vehicle control, ZSE, AgNO_3_, and ZS-Ag-NPs treated adipocytes condition media supplemented HUVECs on cell morphology by light microscopy (**a**), PI Staining for nuclear damage (**b**) and JC-1 staining for mitochondrial membrane potential (**c**) after 48 h.

Figure 7a shows the light microscopic images for morphological changes and microtubule developments in condition media in the vehicle control, AgNO_3_, ZSE, and ZS-Ag-NPs treated adipocyte condition media supplemented HUVECs after 48 h. In Figure 7b, PI staining of ZS-Ag-NPs treated HUVECs showed spherical-shaped nuclei without any nuclear condensation compared to AgNO_3_ or ZSE treated HUVECs. The JC-1 staining of ZS-Ag-NPs treated HUVECs revealed that 62% of negatively charged mitochondria was converted the green-colored lipophilic cationic JC-1 to red-colored J-aggregates; versus 43% and <4% for ZSE and AgNO_3_ treated HUVECs (Figure 7c). 

PI staining; condition media (CM) of the vehicle control-treated HUVECs shows irregularly shaped nuclei with stress cells. CM of AgNO_3_ alone treated HUVECs shows less nuclear content and pyknosis morphology of nuclei. CM of ZS-Ag-NPs treated HUVECs shows clear and round-shaped normal nuclei with no signs of shrinkage, pyknosis, or apoptosis. CM of ZS-Ag-NPs shows better nuclear shape compared to CM of ZES group. 

JC-1 staining, ZS-Ag-NPs treated adipocytes show high J-aggregates, representing active mitochondria (high MMP, Δψ_m_) when compared to ZES or other groups.

#### 3.9.2. Quantification of Gene Expression Levels in HUVECs

**Figure 8:**
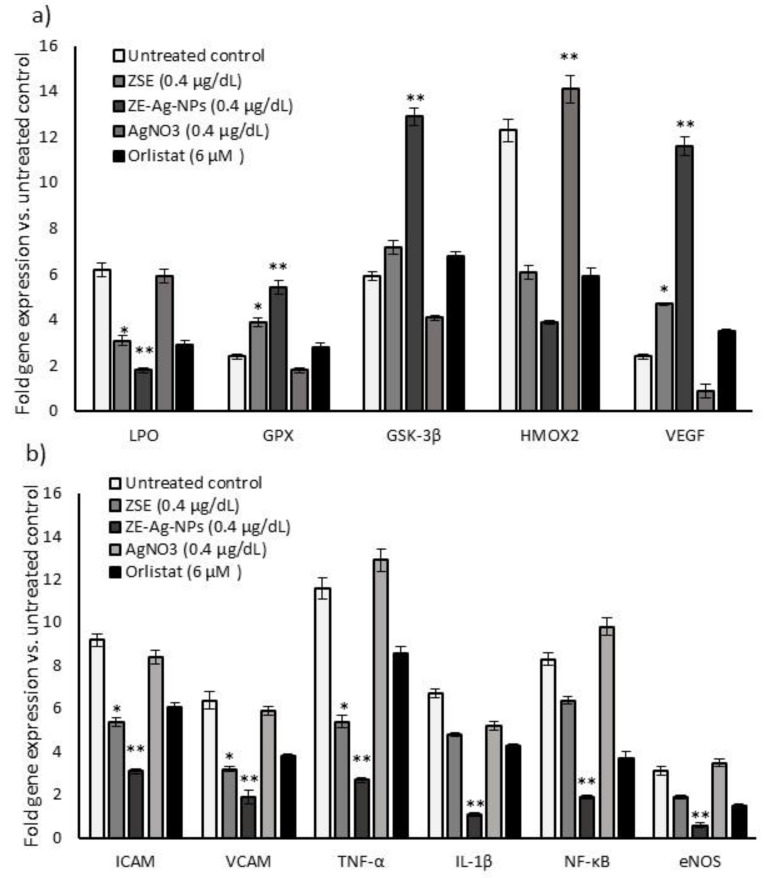
Effect of the vehicle control, ZSE, AgNO_3_, and ZS-Ag-NPs treated adipocytes condition media supplemented HUVECs on oxidative stress and antioxidant (**a**), vascular cell inflammation, and angiogenesis (**b**) related gene expression levels after 48 h. Each value is a mean ± SD (*n* = 6). * significant at *p* ≤ 0.05 and ** highly significant at *p* ≤ 0.001, by comparison with the vehicle control.

Oxidative stress (LPO, GPX, GSK-3β, and HMOX2) and vascular inflammation (ICAM, VCAM, TNF-α, IL-1β, NF-κB, eNOS) related and vascular endothelial cell growth factor (VEGF) mRNA expression levels in the vehicle control, AgNO_3_, ZSE, and ZS-Ag-NPs treated HUVECs for 48 h were quantified and results are shown in Figure 8. The vehicle control and AgNO_3_ treated HUVECs showed significantly (*p* ≤ 0.001) increased levels of LPO, HMOX2, VCAM, eNOS, ICAM, NF-kB, IL-1β, and TNF-α and decreased expression levels of GPX, GSK-3β, and VEGF. ZS-Ag-NPs treated HUVECs revealed a decrease (*p* ≤ 0.001) in all the tested vascular inflammation and oxidative stress-related mRNA expressions when compared to ZSE treated HUVECs. Interestingly, the VEGF expression in ZS-Ag-NPs treated HUVECs was significantly increased by 2.9 folds. The VEGF expression was not identified in the vehicle control or AgNO_3_ treated HUVECs.

## 4. Discussion

Metal nanoparticles, especially silver nanoparticles (AgNPs) have been extensively used in the construction and delivery of active ingredients as nano-carrier systems [50]. AgNPs carry a greater scientific interest and widespread application for the active ingredients delivery vehicles, diagnosis, and therapy. This is due to their high biocompatibility, membrane permeability, surface properties, less toxicity, and broad spectrum antimicrobial activity [11]. Bioresources have been popularly used in the synthesis of greener and more stable silver nanoparticles. Green synthesis of silver nanoparticles is not only biocompatible, degradable, environment friendly, and cost-effective; they mainly act as the stable coating layers which prevent particle aggregation [51]. Many studies have illustrated that plants, microorganisms, and natural polymers are widely used for the green synthesis of AgNPs as the capping and reducing agents [52].

Bioactive ingredients isolated from traditional medicinal plants with lipolytic potentials, with no side effects, have been considered a lot. The GC-MS profile of *Ziziphus spina-christi* root methanol extract contained considerable amounts of indole-3-carboxylic acid, 5-methoxy-2-methyl-1-(3-methylphenyl)-, ethyl ester in the GC-MS profile of *Ziziphus spina-christi* methanol root extract. Numerous derivatives of this compound have biological and pharmacological activities, such as antiviral activity (6-bromo-5-methoxy-indole-3-carboxylic acid [53,54], anxiolytic and anticonvulsant activities (9H-Pyrido[3,4-b]indole-3-carboxylic acid, 4-(methoxymethyl)-6-(phenylmethoxy)-, 1-methylethyl ester (abecarnil) [54], and anticancer, antibacterial, and anti-inflammatory activities [55]. Indole-3-carboxylic acid has a mediator role in plant resistance against necrotrophic pathogens [31]. The indole-3-carbinol is found to decrease body weight and fat accumulation and infiltrated macrophages in epididymal adipose tissue in mice. It also improves glucose tolerance and modulated expression of adipokines and lipogenic-associated gene products, including acetyl coenzyme A carboxylase and peroxisome proliferator-activated receptor-γ [56]. Moreover, some derivatives of phenol, 2,2′-methylenebis[6-(1,1-dimethylethyl)-4-methyl show antimicrobial, antioxidant, and anticancer activities [22,57]. 3H-[1,3,4]Oxadiazole-2-thione, 5-(4,6-dimethylpyrimidin-2-ylsulfanylmethyl), has derivatives synthesized from oxadiazole-2-thione are known as anticancer agents and tubulin polymerization inhibitors [28], and nucleotide pyrophosphatases/phosphodiesterases 1 inhibitors [29]. The flavonol, Kaempferol, has antioxidant, anti-inflammatory, antimicrobial, cardioprotective, and neuroprotective effects [27]. Dibenzo(b,d)pyran derivative of cannabinol has potential immunosuppressive and anti-inflammatory activities and shows a partial agonist at the CB1 and CB2 receptors [25,26]. The compound 3,3-Diphenyl-1-indanone has an indanone moiety, which is used as an intermediate in the synthesis of many types of medicinally important molecules. For instance, it is a precursor moiety of donepezil (IV), an acetylcholinesterase inhibitor that has been approved by the US Food and Drug Administration for the treatment of Alzheimer’s disease [32]. Some 2-benzylidene-1-indanone derivatives have anti-inflammatory effects against acute lung injury [33]. Synthesized 1,4- Phthalazinedione derivatives are found to display a vasorelaxant activity [34] and antibacterial activity [35]. We synthesized *Ziziphus spina-christi* (Jujube) root-loaded silver oxide nanoparticles (ZS-Ag-NPs) with a minimal nanometer size and uniform dispersion, which was confirmed by Zetasizer, TEM, FT-IR, and XRD pattern analyses. Such successful synthesis of silver oxide nanoparticles, with potential biological activities, has been mentioned earlier [33,38,48]. As mentioned above, *Ziziphus spina-christi* (Jujube) root extract contained some phenolic compounds, such as Kaempferol. Phenolics play significant roles in the reduction and stabilization of synthesized metal nanoparticles. They mediate redox reactions by donating electrons to form quinones [58,59]. Rao et al. [59] have reported on the synthesis of gold nanoparticles using the leaf extract of *Cacumen platycladi* and suggested the involvement of flavonoids and reducing sugars as the reducing and capping agents. The role of compounds, such as tannins, eugenol, and flavonoids in the reduction of Ag+ ions to Ag^0^ by the leaf extracts of guava (*Psidium guajava*), have also been reported [60]. It was found that amino acids and proteins have roles in the stabilization of metal nanoparticles [59]. Bioactive compounds, such as ascorbic acid, citric acid, cyclic peptide, ellagic acid, epicatechin gallate, euphol, galangin, gallic acid, phyllanthin, retinoic acid, sorbic acid, and the aflavin have been identified as responsible for the biogenic synthesis of silver nanoparticles. Due to their greater biocompatibility, and applicability, the plant extract derived silver nanoparticles have shown superior antioxidant, anticancer, and antimicrobial activities against clinically isolated pathogens, including multi drug resistant and yeast pathogens [61].

Most interestingly, ZS-Ag-NPs did not produce significant toxicity in adipocytes and human vascular endothelial smooth muscle cells compared to AgNO_3_ alone.

Excessive food intake and diminished energy expenditure resulted in obesity—the most common nutritional disorder. Adipogenesis and lipid accumulation are a complex, interrelated cascade, and it is initiated by the key adipocyte differentiation determinants, mainly, peroxisome proliferator-activated receptor-γ2 (PPARγ2) and CCAAT/enhancer-binding protein-α (C/EBP-α). After terminal differentiation, lipid accumulation was achieved by activating transcription of the gene encoding the fatty acid-binding protein aP2 and sterol-regulatory element-binding protein 1c (SREBP1c) [62]. Further, the activation of these adipogenic factors stimulates lipogenesis and adipogenesis via adiponectin, leptin, glucose transporter-4 (GLUT-4), fatty acid synthase (fas), and adipocyte binding protein (aP2) gene expression [63]. We found that ZS-Ag-NPs treated to maturing adipocyte shown decreased lipid content and increased mitochondrial membrane potential (Δψm); but this effect has not been found in ZSE or AgNO3 alone treated maturing adipocyte after 14 days.

Visceral obesity is strongly associated with a higher risk for the development of metabolic and cardiovascular diseases in human beings [64,65]. Pathophysiology of vascular diseases associated with adipocyte hypertrophy and hyperplasia, specifically perivascular adipocytes might play a major role in obesity because of neovascularization around fat depots [66]. Hypertrophic adipocytes secrete adipokines, which might provide a connection between obesity and vascular complications [67]. In this context, matured adipocyte-secreted adipokines supplemented with oleic acid (OA) noticed to increase the vascular smooth muscle cell proliferation via the induction of iNOS expression, NO production, and pro-inflammatory signaling [68]. Many obesity drugs are found to have adverse side effects, such as pulmonary hypertension, heart diseases, and strokes, which have been later withdrawn from the markets [69]. Recently, the discover of new drugs that are effective in preventing and treating obesity and associated metabolic syndromes, such as type 2 diabetes, draw researcher attentions.

In this study, we used ZS-Ag-NPs treated adipokines (condition media) to treat HUVECs for 48 h, then the influence on angiogenesis or immunomodulatory stimulation was analyzed. The results confirmed that untreated adipocyte condition media (CM) showed HUVEC hyperproliferation and nuclear cellular damage. Simultaneously, ZS-Ag-NPs treated CM did not produce nuclear damage or chromatin condensation to HUVECs, which was confirmed by the PI staining analysis. It is found that that VSMC proliferation can be stimulated by the adipocyte-conditioned medium (CM) [64]. We found that ZS-Ag-NPs treated adipokines effectively inhibited LPO, eNOS, and HO in vascular smooth muscle cells associated with oxidative stress. In addition, HUVECs pro-inflammatory conditioning associated mRNA expressions of ICAM, VCAM, TNF-α, IL-1β, NF-κB decreased two folds in ZS-Ag-NPs treated adipokines. Moreover, mRNA expressions of the vascular endothelial cell growth factor (VEGF) were higher in ZS-Ag-NPs compared with the mRNA expressions in ZSE and vehicle control groups. The anti-inflammatory potential of omentin was found due to the inhibition of TNF-α-induced superoxide production in vascular smooth muscle cells [70].

In conclusion, ZS-Ag-NPs effectively control the adipocyte lipid accumulation and enhance mitochondrial fatty acid oxidation and energy production. ZS-Ag-NPs treated adipokines decreased the oxidative stress and pro-inflammatory cytokines, thus effectively improve smooth muscle cells function and decreased vascular diseases. Increased VEGF level effectively stimulates angiogenesis, which supports antiageing and neovascularization. Overall, silver nanoparticles synthesized using *Ziziphus spina-christi* (Jujube) root extract potentially inhibited obesity progressions and associated vascular complications.

## Figures and Tables

**Table 1:** GC-MS composition of *Ziziphus spins-christi* methanol root extract.

No	RT (min)	Peak Area (%)	Compound Name	Molecular Formula	Molecular Weight (g/mol)	Compound Nature	Bioactivity
1	9.707	5.15	Undecane	C_11_H_24_	156.31	Alkane	Alarm pheromone of the ant *Componotus obscuripes* [18].
2	18.054	9.28	2,4,6-Cycloheptatrien-1-one	C_7_H_6_O	106.12	Cyclic aliphatic ketone	Troponoid derivatives have antibacterial, antifungal, insecticidal, antimalarial, antitumor, anti-ischemic, iron chelating, and the inhibitory activity against polyphenol oxidase activity [19].
			2-Coumaranone	C_8_H_6_O_2_	134.13	Benzofurn ketone	Coumaranone derivatives have pharmaceutical activities against different biological targets [20,21].
3	18.348	18.04	Benzeneacetonitrile, 4-hydroxy-	C_8_H_7_NO	133.15	Aromatic cyanide	Not reported
4	25.654	8.85	2-Propenal, 3-(2-furanyl)-	C_7_H_6_O_2_	122.12	Organoheterocyclic compound	Not reported
5	40.460	5.22	Phenol, 2,2′-methylenebis[6-(1,1-dimethylethyl)-4-methyl	C_23_H_32_O_2_	340.50	Aromatic organic compound	Derivatives are potent antimicrobial, antioxidant and anti-cancer agents [22,23].
6	41.626	2.73	Benzene, 1,2,4-trichloro-5-nitro-	C_6_H_2_Cl_3_NO_2_	226.40	Aromatic compound	Not reported
			1H-Inden-1-one, 2,3-diphenyl-	C_21_H_14_O	282.30	Heterocyclic aromatic ketone	Derivatives (Phytoalexins) are antimicrobial agents [24].
7	43.614	3.41	Cannabinol	C_21_H_26_O_2_	310.40	Dibenzo(b,d)pyran derivative	Immunosuppressive and anti-inflammatory activities, agonist at the CB_1_ and CB_2_ receptors [25,26].
8	43.706	3.04	Kaempferol	C_15_H_10_O_6_	286.24	Flavonoid	Antioxidant, anti-inflammatory, antimicrobial, cardio-, and neuroprotective effects [27].
9	46.223	4.44	3H-[1,3,4]Oxadiazole-2-thione, 5-(4,6-dimethylpyrimidin-2-ylsulfanylmethyl)-	C_9_H_10_N_4_OS_2_	254.30	An oxadiazole-2-thione derivative	Derivatives showed anticancer and tubulin polymerization inhibitor [28] and nucleotide pyrophosphatases/phosphodiesterases 1 inhibitors [29].
10	47.011	19.02	Indole-3-carboxylic acid, 5-methoxy-2-methyl-1-(3-methylphenyl)-, ethyl ester	C_20_H_25_N_3_O	323.40	Heterocyclic benzopyrrole	Anticonvulsant, anticancer, antibacterial, and anti-inflammatory, antitubercular, antimalarial activities [30]. Plant resistance mediator against necrotrophic pathogens [31].
11	47.943	9.16	3,3-Diphenyl-1-indanone	C_21_H_16_O	284.30	Heterocyclic aromatic ketone	1-indanone derivatives have anti-inflammatory effects [32,33].
			1,4- Phthalazinedione, 2,3-dihydro-6-nitro-	C_8_H_5_N_3_O_4_	207.14	Heterocyclic organic compound	Derivatives have vasorelaxant activity [34] and antibacterial activity [35].

## Data Availability

The data presented in this study are available in the article or supplementary materials.

## Supplementary Materials

The following are available online at https://www.mdpi.com/article/10.3390/nano11102563/s1. Figure S1. GC-MS composition of *Ziziphus spina-christi* root methanol extract. Figure S2a. Prepared nanoparticles with increasing concentration of AgNo_3_; Figure S2b. UV-visible spectrum data; Figure S2c. FT-IR spectrum data. Figure S3a. XRD patterns of AgNO_3_; Figure S3b. XRD patterns of 5 mM AgNPs; Figure S3c. XRD patterns of 10 mM AgNPs. Figure S4a. Size distribution by intensity of AgNO_3_; Figure S4b. XRD Size distribution by intensity of 5 mM AgNPs; Figure S4c. Size distribution by intensity of 10 mM AgNPs.
