# Supplementary figures and images for: Synthesis of Ziziphus spina-christi (Jujube) Root Methanol Extract Loaded Functionalized Silver Nanoparticle (ZS-Ag-NPs); Physiochemical Characterization and Effect of ZS-Ag-NPs on Adipocyte Maturation, Adipokine and Vascular Smooth Muscle Cell Interaction
Source: Nanomaterials (Basel). 2021 Sep 29;11(10):2563. doi: 10.3390/nano11102563 (PMC8539395; doi:10.3390/nano11102563)

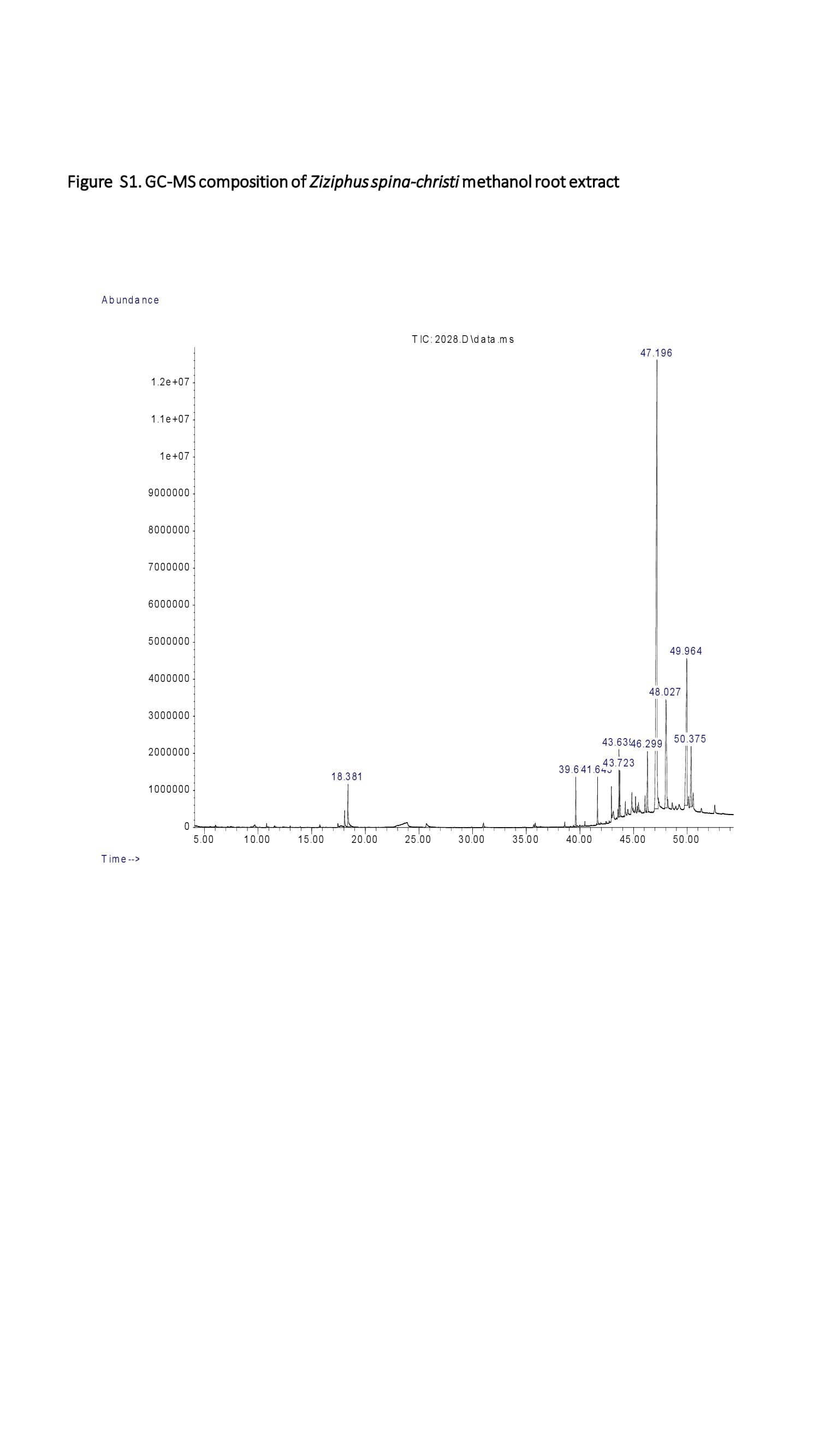

Supplement: Supplementary file 1 [file nanomaterials-11-02563-s001.zip › Supplementary figures_00001.jpg]

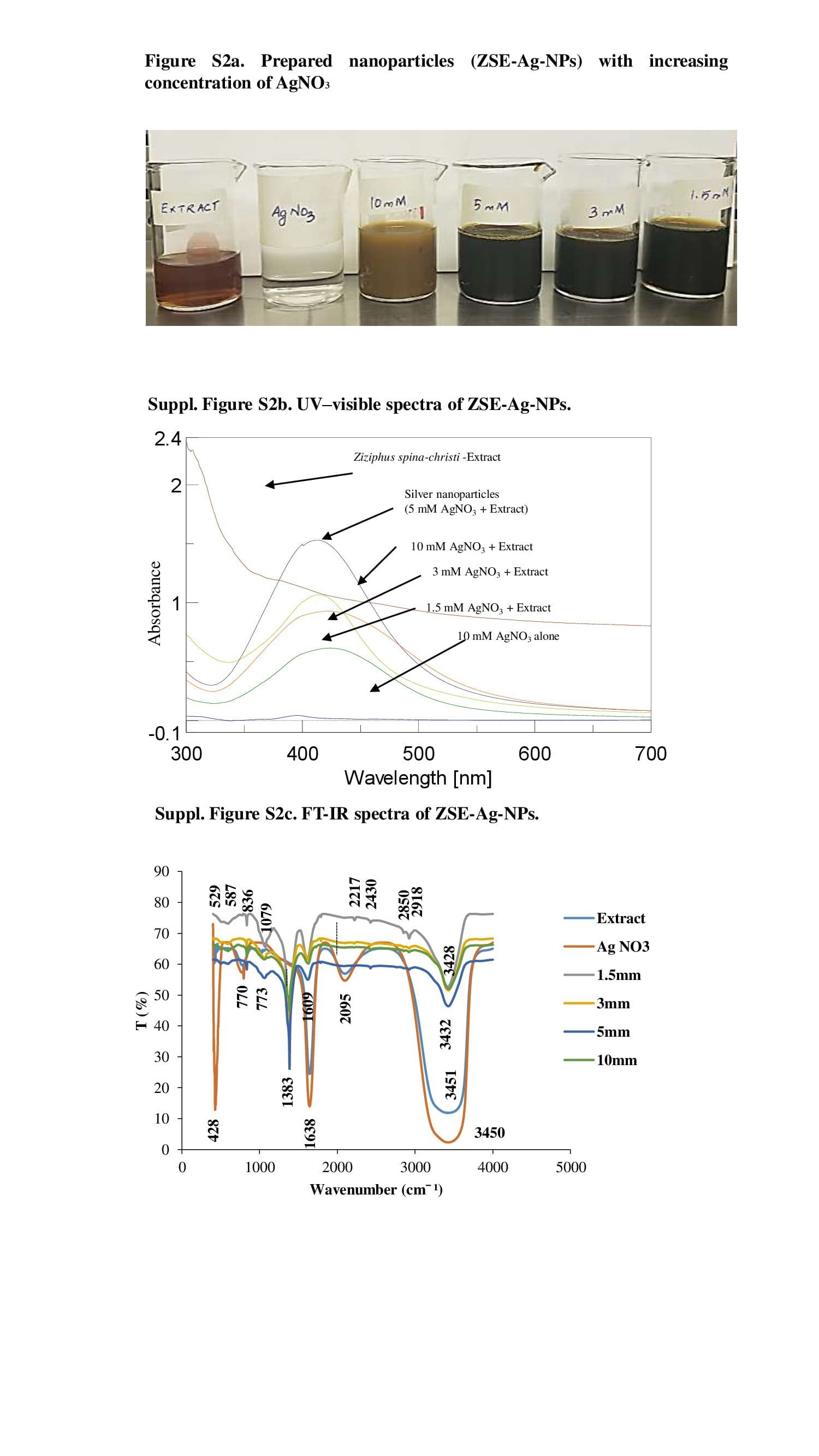

Supplement: Supplementary file 1 [file nanomaterials-11-02563-s001.zip › Supplementary figures_00002.jpg]

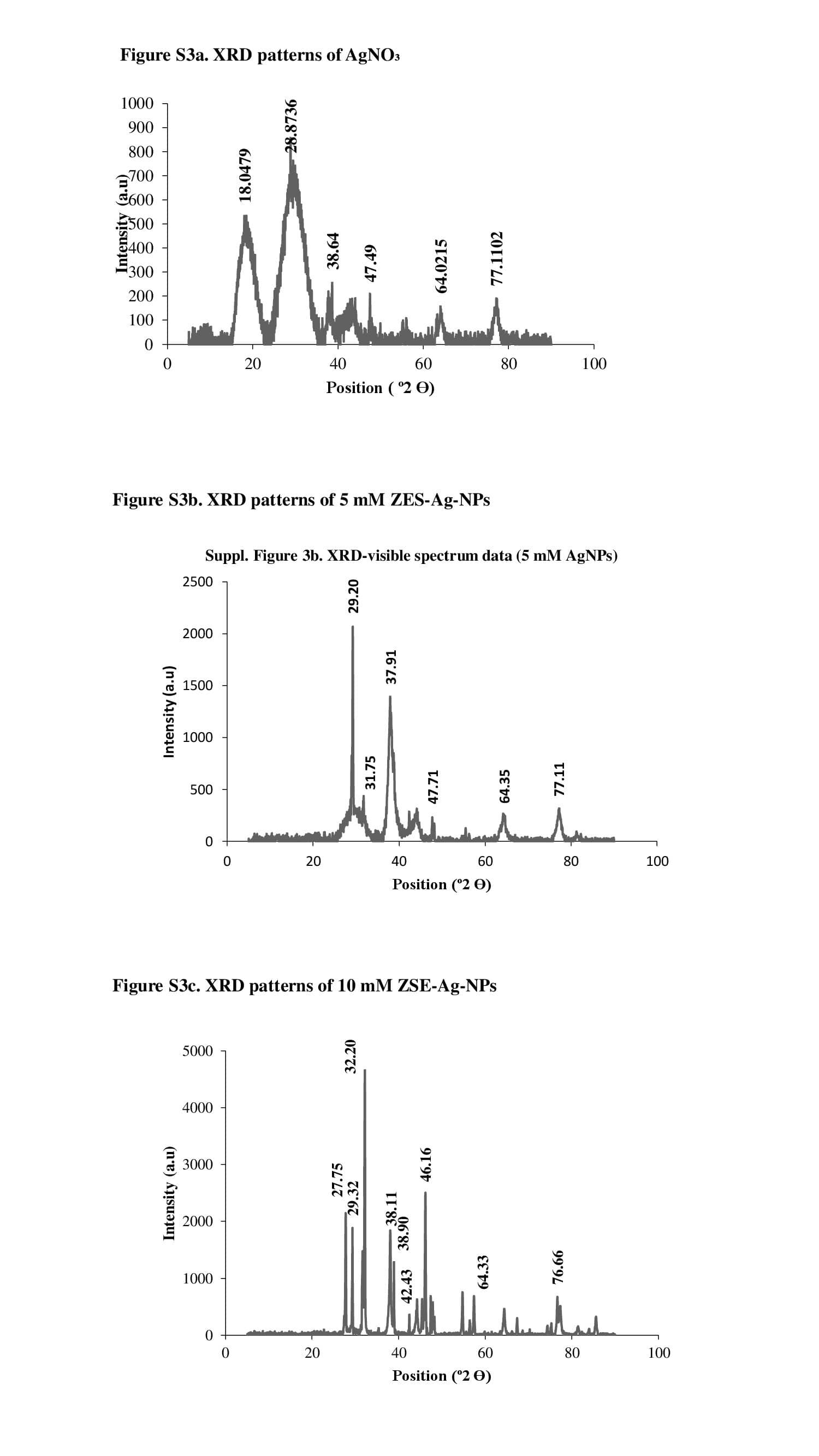

Supplement: Supplementary file 1 [file nanomaterials-11-02563-s001.zip › Supplementary figures_00003.jpg]

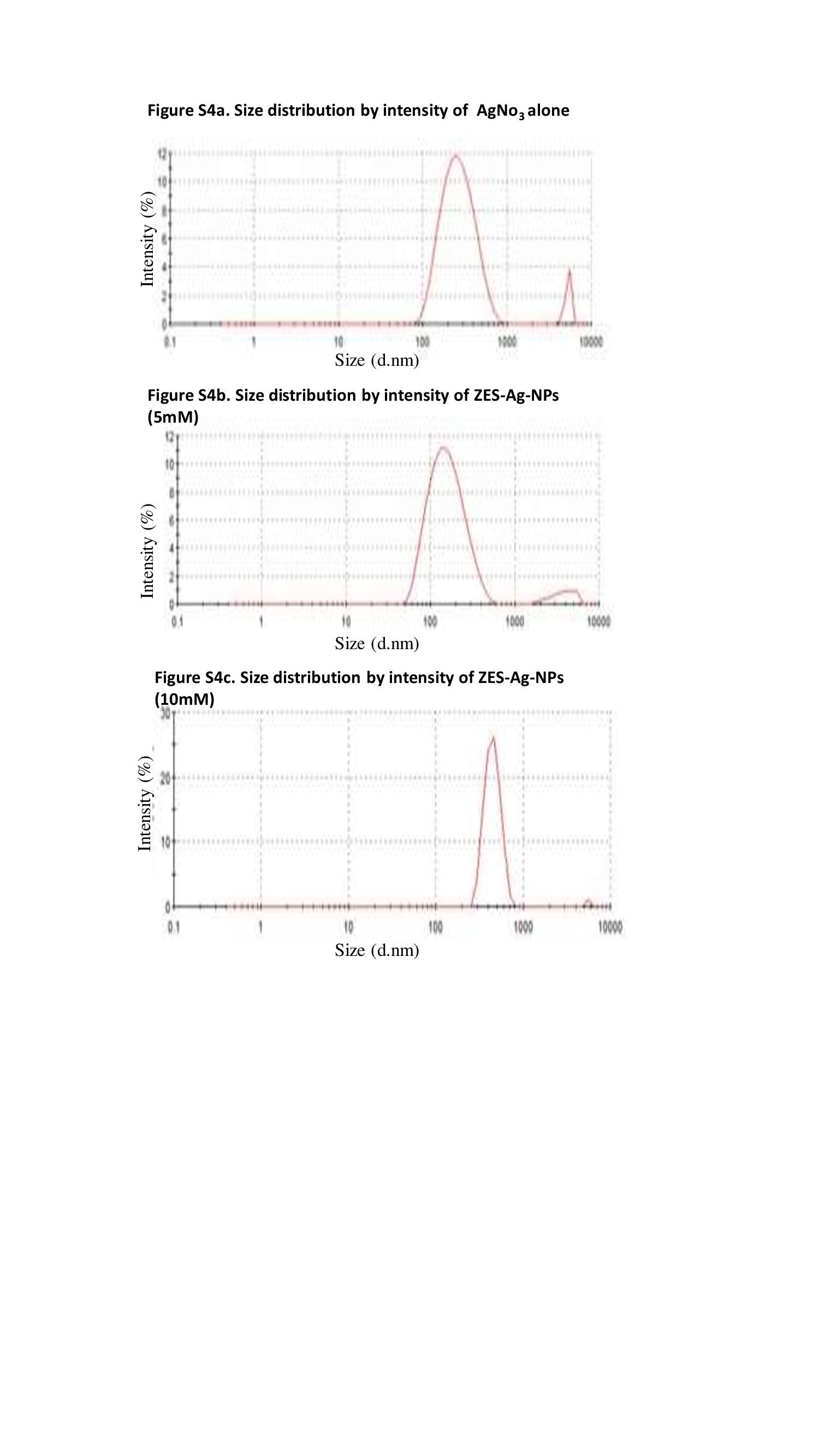

Supplement: Supplementary file 1 [file nanomaterials-11-02563-s001.zip › Supplementary figures_00004.jpg]
